# Current conceptual challenges in the study of rhythm processing deficits

**DOI:** 10.3389/fnins.2015.00197

**Published:** 2015-06-08

**Authors:** Pauline Tranchant, Dominique T. Vuvan

**Affiliations:** ^1^International Laboratory for Brain, Music, and Sound Research (BRAMS), Université de MontréalMontreal, QC, Canada; ^2^Centre for Research of Brain, Language, and MusicMontreal, QC, Canada

**Keywords:** rhythm, beat, meter, beat deafness, auditory-motor mapping, dysrhythmia, sensorimotor synchronization, poor synchronization

## Abstract

Interest in the study of rhythm processing deficits (RPD) is currently growing in the cognitive neuroscience community, as this type of investigation constitutes a powerful tool for the understanding of normal rhythm processing. Because this field is in its infancy, it still lacks a common conceptual vocabulary to facilitate effective communication between different researchers and research groups. In this commentary, we provide a brief review of recent reports of RPD through the lens of one important empirical issue: the method by which beat perception is measured, and the consequences of method selection for the researcher's ability to specify which mechanisms are impaired in RPD. This critical reading advocates for the importance of matching measurement tools to the putative neurocognitive mechanisms under study, and reveals the need for effective and specific assessments of the different aspects of rhythm perception and synchronization.

Scientific interest in rhythm processing in music has exploded in the last decade. This has been accompanied by growing interest in rhythm processing deficits (RPD)^1^^1^, which may serve as a powerful tool for the investigation of the normal processing of rhythm. Because the study of RPD is in its infancy, the empirical approach to its study has been inconsistent, particularly with respect to the methods used to measure beat perception. These inconsistencies invoke challenges for RPD researchers, particularly concerning the identification of which rhythm-related mechanisms (beat perception or synchronization, for instance) are impaired, and how the different mechanisms might interact. In this commentary, we discuss how these tensions are exemplified in three recently published reports (Phillips-Silver et al., 2011; Sowiński and Dalla Bella, 2013; Launay et al., 2014).

One constant across these studies is their use of the Montreal Battery of Evaluation of Amusia (MBEA; Peretz et al., 2003) to assess rhythm perception. The MBEA has been designed to diagnose music processing disorders along two distinct processing pathways; one related to melodic organization and the other related to temporal organization (the rhythm). This separation was motivated by neuropsychological dissociations (see Peretz, 2013 for a recent review; see Phillips-Silver et al., 2013 for a recent empirical report). Within the temporal dimension the assessment of rhythm perception is divided into two subtests: the rhythm test and the metric test. Like the MBEA's division of pitch and temporal processing, this division was motivated by previous neuropsychological dissociations (Fries and Swihart, 1990; Peretz, 1990; Liégeois-Chauvel et al., 1998) suggesting two separable mechanisms for the processing of musical rhythm: the tendency to cluster the sounded events that constitute a rhythm into figural patterns according to temporal proximity (grouping) and the emergence of regularly recurring psychological events in response to a rhythm (beat).

In the rhythm test, participants have to judge whether two short piano excerpts are the same or different, with different trials containing alterations produced by manipulating the durations of two adjacent tones, so that the rhythm is changed but the total number of sounds and the meter are preserved. Importantly, although beat perception may be helpful to perform this task in normal participants, it is not necessary. Specifically, the comparison of the pattern of durations in each sequence to be judged is sufficient for task success. In contrast, the metric test targets beat perception by asking participants to judge whether short piano excerpts are marches (binary metrical organization: alternation of a strong beat and a weak beat) or waltzes (ternary metrical organization: one strong beat followed by two weak beats). Interestingly, like for the rhythm test, an alternative strategy that does not tap beat perception is possible. Specifically, the perception of the acoustic accents used to mark strong beats and the counting of the intervening events would be sufficient for task success.

The first study of congenital RPD (Phillips-Silver et al., 2011) reported the case of a university student, Mathieu, unable to synchronize simple whole-body movement (bouncing) with a musical beat despite preserved cognitive, motor, and pitch-related musical abilities. Mathieu performed comparably to controls on the MBEA rhythm test, but performed poorly on the metric test. The authors thus proposed that “an inability to detect an underlying beat” may be responsible for his disorder, which they labeled “beat deafness.”

In the second paper, Sowiński and Dalla Bella (2013) reported four participants who exhibit poor synchronization to a beat. Rhythmic perception was assessed with both the rhythm test of the MBEA and with an “anisochrony detection task.” Two cases (S2 and S9) performed poorly at an anisochrony detection task (henceforth ADT) and the authors concluded that they were thus comparable to Mathieu (i.e., synchronization deficit due to perception deficit). We argue that this conclusion may not be valid for two reasons. First, although Phillips-Silver et al. (2011) proposed the perceptual origin of Mathieu's disorder on the basis of his poor performance on the metric test of the MBEA, Sowiński and Dalla Bella (2013) did not report S2 and S9's performance on this test. The reason for this is that these authors' sample performed with high variability on the metric test, which might compromise the use of 2 SD below the mean as the impairment threshold. However, this does not invalidate the use of the MBEA metric test as a measure of beat perception. Rather, this indicates that a different threshold that takes this high variability into account should be sought, particularly because the distribution of performance on this task is non-normal (skewed to the left, as indicated by unpublished norms from our group with *n* = 432). Second, like the MBEA rhythm and meter tests, beat perception may facilitate performance but it is not necessary to succeed at the ADT. Indeed, it can be performed by comparing the durational values of adjacent inter-tone-intervals, and for the musical sequences, by noting the acoustic cue produced by the jittered onsets of the high and low voices on anisochronic trials (these stimuli were acquired through personal correspondence with Sowiński and Dalla Bella, 2013). This proposition is consistent with work by Grahn and McAuley (2009) showing that strong and weak beat perceivers do not differ in their ability to judge whether the final interval in a metronome sequence is different from the intervals preceding it.

Sowiñski and Dalla Bella further concluded that the synchronization deficits observed in two additional cases (S1 and S5) might be due to a disorder of auditory-motor mapping rather than a beat perception disorder. We argue that caution should be exercised before making this claim. This conclusion can only be reached if an impairment of beat perception has been excluded, which is not the case because, as noted above, both the MBEA rhythm test and anisochrony detection tasks can be performed without beat perception.

Finally, Launay et al. (2014) screened participants for rhythm perception impairments using the MBEA rhythm test and showed that three individuals identified through this procedure exhibited impaired synchronization when tapping to a beat. The authors named this condition “dysrhythmia.” These authors inferred that the deficit observed in their three impaired participants “seems to lie specifically in extracting the correct (intended) meter from non-isochronous metrical rhythms,” despite the fact that, as discussed above, the capacity to perceive a beat is not necessary to perform the MBEA rhythm test. Therefore, the locus of the dysrhythmic deficit thus remains unclear. In particular, a clear model for how poor beat perception and consequently poor synchronization might result from poor temporal duration perception abilities is lacking. For instance, the perception of temporal duration may be necessary for beat extraction and hence for synchronization to the beat. Alternatively, if duration perception is dissociable from beat perception, or if perception is dissociable from synchronization, then the perception of interval duration perception may be unrelated to a beat synchronization deficit. Regardless, without the adequate measurement of beat perception, a strong conclusion cannot be reached about the source of the observed synchronization deficit.

## Conclusions

We anticipate an explosion of studies on rhythm deficits in the coming years, as was the case for pitch-related deficits after the original introduction of the MBEA (Peretz et al., 2003). A primary goal for researchers in the years to come will be to find ways to clarify the origin of such deficits, driving the development of myriad research questions. For example, which temporal mechanisms are specifically impaired? Is the deficit purely perceptual or does it involve impaired sensorimotor coupling? Is the disorder music-specific or domain-general? The ability of such research to illuminate important research questions regarding timing behavior depends critically on the careful use of measurement tools to assess the specific mechanisms hypothesized to underlie various components of this behavior. One tool that has much potential for the measurement of beat perception in RPD is the Beat Alignment Test (BAT; Iversen and Patel, 2008), which tests beat perception through the judgment of whether or not an isochronous train of beeps superimposed upon a musical extract sounds “on the beat” or not. The BAT is particularly promising because of the lack of obvious strategies, other than beat perception, that can be deployed to perform this task. Two further potentially interesting tools are the Battery for the Assessment of Auditory Sensorimotor and Timing Abilities (BAASTA; Benoit et al., 2014) and the Harvard Beat Assessment Test (H-BAT; Fujii and Schlaug, 2013). The BAASTA provides a package of tests including the BAT, anisochrony detection, and a task that explicitly assesses duration discrimination in the absence of a beat. The H-BAT assesses beat perception and production using both musical excerpts and psychophysically-controlled woodblock stimuli. In sum, the use of untapped tools, such as the BAT, the BAASTA, and the H-BAT, as well as the development of novel tools for the measurement of beat perception must be a central aim of RPD research for the near future.

### Conflict of interest statement

The authors declare that the research was conducted in the absence of any commercial or financial relationships that could be construed as a potential conflict of interest.

## Footnotes

^1^This paper will focus on rhythm processing deficits that neither result from a brain injury nor are secondary to a neurolological/psychiatric condition (e.g., Parkinson's disease, schizophrenia, etc.; see Allman and Meck, 2011).
